# Research on Three-Phase Asynchronous Motor Fault Diagnosis Based on Multiscale Weibull Dispersion Entropy

**DOI:** 10.3390/e25101446

**Published:** 2023-10-13

**Authors:** Fengyun Xie, Enguang Sun, Shengtong Zhou, Jiandong Shang, Yang Wang, Qiuyang Fan

**Affiliations:** 1 School of Mechanical Electrical and Vehicle Engineering, East China Jiaotong University, Nanchang 330013, China; sngzm999@163.com (E.S.); zhoust@ecjtu.edu.cn (S.Z.); jdshang1997@163.com (J.S.); wwyy0130@163.com (Y.W.); fqy18532671715@163.com (Q.F.); 2 State Key Laboratory of Performance Monitoring Protecting of Rail Transit Infrastructure, East China Jiaotong University, Nanchang 330013, China; 3 Life-Cycle Technology Innovation Center of Intelligent Transportation Equipment, Nanchang 330013, China

**Keywords:** Weibull distribution, multiscale dispersion entropy, particle swarm optimization, support vector machine, fault diagnosis

## Abstract

Three-phase asynchronous motors have a wide range of applications in the machinery industry and fault diagnosis aids in the healthy operation of a motor. In order to improve the accuracy and generalization of fault diagnosis in three-phase asynchronous motors, this paper proposes a three-phase asynchronous motor fault diagnosis method based on the combination of multiscale Weibull dispersive entropy (WB-MDE) and particle swarm optimization–support vector machine (PSO-SVM). Firstly, the Weibull distribution (WB) is used to linearize and smooth the vibration signals to obtain sharper information about the motor state. Secondly, the quantitative features of the regularity and orderliness of a given sequence are extracted using multiscale dispersion entropy (MDE). Then, a support vector machine (SVM) is used to construct a classifier, the parameters are optimized via the particle swarm optimization (PSO) algorithm, and the extracted feature vectors are fed into the optimized SVM model for classification and recognition. Finally, the accuracy and generalization of the model proposed in this paper are tested by adding raw data with Gaussian white noise with different signal-to-noise ratios and the CHIST-ERA SOON public dataset. This paper builds a three-phase asynchronous motor vibration signal experimental platform, through a piezoelectric acceleration sensor to discern the four states of the motor data, to verify the effectiveness of the proposed method. The accuracy of the collected data using the WB-MDE method proposed in this paper for feature extraction and the extracted features using the optimization of the PSO-SVM method for fault classification and identification is 100%. Additionally, the proposed model is tested for noise resistance and generalization. Finally, the superiority of the present method is verified through experiments as well as noise immunity and generalization tests.

## 1. Introduction

Three-phase asynchronous motors, as an efficient, economical, and practical type of motor, are vital for the development and operation of various industries [1]. In industrial production, transport, construction, and household appliance industries, they drive various equipment items and machinery, such as high-speed trains, fans, pumps, compressors, conveyor belts, machine tools, etc., providing the necessary power for industrial production and life [2]. Three-phase asynchronous motors often run in alternating electromagnetic fields and high load conditions; however, failure is inevitable, which causes a great threat to the country, society, personal life, and property safety [3]. Therefore, the health monitoring and intelligent fault diagnosis of three-phase asynchronous motors are of great significance.

In the field of asynchronous motors, common fault diagnosis methods include the following: 

Vibration analysis: By analyzing and processing the vibration signals of the motor, it is possible to determine whether there are any abnormal vibration conditions in the motor, such as bearing failure, imbalance, mechanical looseness, etc. 

Temperature monitoring: By monitoring the temperature changes of the motor, it is possible to determine whether there are problems such as overheating and overloading of the motor, thus predicting the life and health of the motor. 

Insulation Detection: By measuring the insulation resistance of the motor, it is possible to determine whether there is insulation damage or leakage in the motor, thus predicting the safety and stability of the motor. 

Current analysis: By analyzing the current waveform and spectrum of the motor, it is possible to determine whether there are faults such as a current imbalance, short circuits between phases, disconnection, and so on, so as to judge the motor’s operation status and health condition. 

Sound analysis: By analyzing the sound signals generated when the motor is running, it is possible to determine whether there are abnormal noises, vibrations, and other problems with the motor so as to judge the motor’s operating status and health condition. 

Motor parameter monitoring: Through real-time monitoring and analysis of the motor’s current, voltage, power, and other parameters, it can be determined whether there are abnormal working conditions and fault conditions of the motor so as to take timely and appropriate maintenance measures.

The superiority of different health monitoring and intelligent fault diagnosis methods depends on the extraction of features and the selection of classifiers [4]. In terms of feature extraction, entropy is a measure that describes the complexity and uncertainty of a signal and is used to measure the amount of information in a signal [5]. Entropy can provide global features of a signal that are not limited by a specific time or frequency range, while traditional time–frequency domain features can usually only provide localized information. Entropy is very sensitive to the nonlinear features of a signal, and traditional time–frequency domain features may not be able to capture these nonlinear features efficiently [6]. Entropy is relatively robust to noise (traditional time–frequency domain features are susceptible to noise) and is able to resist the influence of noise to a certain extent and more accurately reflect the characteristics of a signal [7]. Steven M. Pincus proposed approximate entropy [8] from the perspective of measuring the complexity of a signal sequence as a measure of the magnitude of the probability of generating a new pattern in the signal; however, this approach involves a comparison with its own vectors, which is incompatible with the new information viewpoint and can be biased. Richman and Moornan proposed sample entropy [9] which does not depend on the length of the data and has better consistency, but it also uses a unit-step function which is more abrupt and lacks continuity in the entropy value. Bandt and Pompe proposed arrangement entropy [10], which is computationally simple and strongly noise-resistant, but this method suffers from excessive computational complexity and ineffectiveness. Weiting Chen proposed fuzzy entropy [11], a method that quantifies uncertainty but produces a degree of discrimination that is too small. In terms of classifier selection, Vapnik proposed support vector machines [12]; this method is very effective for high-dimensional number spaces but requires the manual extraction of parameters and is not efficient. Kennedy and Eberhart proposed particle swarm optimization [13]; this method has few parameters and is simple and easy to implement.

In view of the problems in the above studies, this paper extracts the features of three-phase asynchronous motors using WB and MDE, then classifies and identifies the fault states via PSO and SVM, and, finally, verifies the superiority of this method through experiments, a noise immunity test, and a generalization test.

The contribution of entropy in the field of fault diagnosis is to help diagnosticians determine the cause and location of faults by measuring the uncertainty and the amount of information in a system. In this paper, a three-phase asynchronous motor fault diagnosis method based on the combination of multiscale Weibull dispersion entropy and a particle swarm optimization support vector machine is proposed. The contributions of this paper are as follows:

(1) In terms of theoretical research, for the nonlinear and unsteady characteristics of three-phase asynchronous motor fault signals, traditional feature extraction cannot fully and comprehensively mine the fault information nor obtain more abundant information about the motor operating state. The WB-MDE proposed in this paper is able to linearize and smooth the vibration data and is able to quantify the regularity of the data to obtain sensitive information about the motor faults, which fills in the gap in this area of research.
(2) In terms of noise resistance and generalization, the traditional methods do not have good noise resistance and generalization and cannot be widely applied. The model proposed in this paper has good noise resistance and generalization in three-phase asynchronous motor fault diagnosis. In three-phase asynchronous motor health monitoring and intelligent fault diagnosis, the recognition effect is more stable and has better generalization ability and noise resistance, which means it can be more widely applied in practice.

The rest of this paper is organized as follows: Section 2 introduces the WB-MDE, t-distributed stochastic neighborhood embedding, and PSO-SVM methods and describes the fault diagnosis process. Section 3 introduces the construction of the experimental data acquisition platform and the experimental design and procedure. Section 4 introduces the analysis of the raw data, the WB-MDE parameter sensitivity analysis, the result analysis of different features, and the result analysis of different classifiers. Section 5 describes the noise resistance test and generalization test of the model proposed in this paper. Finally, conclusions are presented in Section 6.

## 2. Introduction of Principles

### 2.1. Multiscale Weibull Dispersion Entropy (WB-MDE)

Multiscale Weibull distribution dispersion entropy (WB-MDE) uses a global search and has better accuracy with nonlinear problems. If the distribution has diversity, it can fit unsteady models and can be scaled up to multiple time scales in order to provide an additional viewing perspective when the time scale is uncertain.

For a time series N:X=$\left\{x_{1},x_{2},\cdot \cdot \cdot ,x_{N}\right\}$ of length *N*, it is first mapped to $y=\left\{y_{1},y_{2},\cdot \cdot \cdot ,y_{N}\right\}$ through the Weibull cumulative distribution function; x and y are vectors and the definition is as follows:

(1) $$y_{j}=\int_{-\infty }^{x_{j}}\frac{\beta }{\eta^{\beta }}t^{\beta -1}e^{-{\left(\frac{t}{\eta }\right)}^{\beta }}dt$$

where j=1,2,⋅⋅⋅,N, β is the shape parameter, η is the scaling factor, and y is mapped to the range $\left[1,2,\cdots c\right]$; β and η are parameters and the definition is as follows:

(2) $$z_{j}^{c}=round(c\cdot y_{j}+0.5)$$

where round is the rounding function and *c* is the number of categories. Calculating the embedding vector $z_{i}^{m,c}$, the definition is as follows:

(3) $$z_{i}^{m,c}=\left\{z_{i}^{c},z_{i+d}^{c},\cdot \cdot \cdot ,z_{i+(m-1)d}^{c}\right\}$$

where i=1,2,⋅⋅⋅,N−(m−1)d and *d* is the delay time. Calculating the dispersion modes, each $z_{i}^{m,c}$ can be mapped to the dispersion mode $\pi_{v_{0}v_{1}\cdot \cdot \cdot v_{m-1}}$. $z_{i}^{c}=v_{0},$ $z_{i+d}^{c}=v_{1},\cdot \cdot \cdot ,z_{i+(m-1)d}^{c}=v_{m-1}$. Since the time series has m points and each mode can take an integer between 1 and c, the time series $z_{i}^{m,c}$ has $c^{m}$ dispersion modes. Calculating the likelihood of each dispersion pattern occurring, the definition is as follows:

(4) $$p(\pi_{v_{0}v_{1}\cdot \cdot \cdot v_{m-1}})=\frac{Number\left\{i\left|i\leq N-(m-1)d,z_{i}^{m,c}hastype\pi_{v_{0}v_{1}\cdot \cdot \cdot v_{m-1}}\right.\right\}}{N-(m-1)d}$$

where *m* is the embedding dimension and Number$\left\{i\left|i\leq N-(m-1)d,z_{i}^{m,c}hastype\pi_{v_{0}v_{1}\cdot \cdot \cdot v_{m-1}}\right.\right\}$ is the number of mappings from $z_{i}^{m,c}$ to $\pi_{v_{0}v_{1}\cdot \cdot \cdot v_{m-1}}$. For a time series u=$\left\{u_{1},u_{2},\cdot \cdot \cdot ,u_{n}\right\}$ of length *n*, with coarse-grained sequences with a scale factor of τ, we have

(5) $$x_{j}^{\tau }=\frac{1}{\tau }\sum_{b=(j-1)\tau +1}^{j\tau }u_{b},1\leq j\leq \left[\frac{L}{\tau }\right]=N$$

where *L* is the length of the data, and the value of the dispersion entropy MDE of the coarse-grained sequence is calculated for each scale factor τ; the definition is as follows:

(6) $$MDispEn(x,m,c,d,\tau )=-\sum_{\pi =1}^{c^{m}}p(\pi_{v_{0}v_{1}\cdot \cdot \cdot v_{m-1}})\cdot \ln(p(\pi_{v_{0}v_{1}\cdot \cdot \cdot v_{m-1}}))$$

### 2.2. t-Distributed Stochastic Neighborhood Embedding (t-SNE)

t-distributed stochastic neighbor embedding (t-SNE) [14] is a dimensionality reduction technique to solve the crowding problem caused by the high dimensionality of the data; it is a more traditional method in unsupervised learning. The original structure of the data can be greatly maintained via this method, and the conversion from high to low dimensionality can be realized [15].

For the distribution of high-dimensional data $X=\{x_{1},x_{2},x_{3},\cdots x_{n}\}$, the joint probability density function of the Gaussian distribution is used; the definition is as follows:

(7) $$p_{ij}=\frac{p_{i\left|j\right.}+p_{j\left|i\right.}}{2N}$$

where *N* is the amount of data and δ is the standard deviation.

$$p_{i\left|j\right.}=\frac{\exp[-{\left\|x_{j}-x_{i}\right\|}^{2}/\left(2\delta_{i}^{2}\right)]}{\sum_{k\neq j}\exp\left[-{\left\|x_{j}-x_{k}\right\|}^{2}/\left(2\delta_{i}^{2}\right)\right]},\;p_{j\left|i\right.}=\frac{\exp[-{\left\|y_{i}-y_{j}\right\|}^{2}/\left(2\delta_{i}^{2}\right)]}{\sum_{k\neq i}\exp\left[-{\left\|y_{i}-y_{k}\right\|}^{2}/\left(2\delta_{i}^{2}\right)\right]}.$$

For the distribution of the reduced dimensional data $Y=\{y_{1},y_{2},y_{3},\cdots y_{m}\}$, the joint probability density function of the t-distribution is used to represent the distribution. The definition is as follows:

(8) $$q_{ij}=\frac{{\left(1+{\left\|y_{i}-y_{j}\right\|}^{2}\right)}^{-1}}{\sum_{k\neq l}{\left(1+{\left\|y_{k}-y_{l}\right\|}^{2}\right)}^{-1}}$$

Based on the joint density function, let the cost function be as follows:

(9) $$c=\sum_{i}\sum_{j}p_{ij}\log\frac{p_{ij}}{q_{ij}}$$

Taking the partial derivative of cost function *c* with respect to $y_{i}$ and obtaining the calculation gradient, the definition is as follows:

(10) $$\frac{\partial c}{\partial y_{i}}=4\sum_{j}\left(p_{ij}-q_{ij}\right)\left(y_{i}-y_{j}\right){\left(1+{\left\|y_{i}-y_{j}\right\|}^{2}\right)}^{-1}$$

The low-dimensional expression is used as the final result, and optimization is sought for the intermediate variable function *c*. The low-dimensional data are continuously updated until the optimal result is reached. The definition is as follows:

(11) $$Y^{t}=Y^{t-1}+\omega \frac{\partial c}{\partial y_{i}}+\alpha \left(t\right)\left(Y^{t-1}+Y^{t-2}\right)$$

where ω is the learning rate and $\alpha \left(t\right)$ is the learning momentum.

### 2.3. Particle Swarm Optimization–Support Vector Machine (PSO-SVM)

Consider a sample set $\left\{(x_{1},y_{1}),(x_{2},y_{2}),\cdot \cdot \cdot ,\right.$$\left.\left(x_{n},y_{n}\right)\right\}$, where $x_{i}\in R^{n},\;y_{i}=\left\{-1,1\right\}$ and *n* is the number of samples. The expression for the linear discriminant function is as follows:

(12) $$f\left(x\right)=w\cdot x+b$$

where w is the weight and *b* is the hyperplane intercept. The categorical hyperplane expression corresponding to the above equation is as follows:

(13) w⋅x+b=0

Normalize $f\left(x\right)$; if any sample satisfies $\left|f\left(x\right)\right|\leq 1$; let the sample closest to the decision surface $\left|f\left(x\right)\right|=1$ satisfy the condition as follows:

(14) $$y_{i}\left(w\cdot x+b\right)-1\geq 0\;i=1,2,\cdot \cdot \cdot ,n$$

The spacing surface distance is $2/\left\|w\right\|$. If the spacing distance is maximized, then $\left\|w\right\|$ is minimized, and for the linearly indistinguishable case, the optimal classification surface is added with the slack variable $\xi_{i}$. The optimal classification surface is as follows:

(15) $$f=\underset{w,b}{\min}\frac{1}{2}{\left\|w\right\|}^{2}+C\sum_{i=1}^{l}\xi_{i}$$

where $\xi_{i}\geq 0$ and *C* is the penalty parameter. For the nonlinear case, the kernel function is added:

(16) $$K(x_{i},x_{j})=\phi \left(x_{i}\right)\cdot \phi \left(x_{j}\right)$$

where ϕ denotes the mapping from the original space to the feature space. Thus, the objective function of nonlinear SVM is as follows:

(17) $$f=\max\sum_{i=1}^{l}\alpha_{i}-\sum_{i}^{l}\sum_{j}^{l}\alpha_{i}\alpha_{i}y_{i}y_{j}K(x_{i},x_{j})\sum_{i=0}^{l}\alpha_{i}y_{i}=0\;0\leq \alpha \leq C;i=1,2,\cdot \cdot \cdot ,l$$

The radial basis kernel function requires only one change in parameter, which is more timeliness, and its mathematical expression is as follows:

(18) $$K(x_{i},x_{j})=\exp\left[-\frac{{\left|x_{i}-x_{j}\right|}^{2}}{\sigma^{2}}\right]$$

The particle swarm optimization algorithm (PSO) finds the optimal solution by iterative updating [16], and its standard formula updating speed $v_{ij}^{k+1}$ and position $x_{ij}^{k+1}$ are as follows:

(19) $$v_{ij}^{k+1}=wv_{ij}^{k}+c_{1}r_{1}\left(p_{ij}^{k}-x_{ij}^{k}\right)+c_{2}r_{2}\left(p_{gj}^{k}-x_{ij}^{k}\right)$$

(20) $$x_{ij}^{k+1}=x_{ij}^{k}+v_{ij}^{k+1}$$

where $c_{1}c_{2}$ is the acceleration factor, and $r_{1},r_{2}\in round$ $\left[0,1\right]$ $v_{ij}^{k},x_{ij}^{k},p_{ij}^{k},p_{gj}^{k}$ are the velocity, position, individual extreme optimal position, and population extreme optimal solution position of the j-dimensional variable of parameter *i* at the *K*th iteration, respectively [17].

### 2.4. Fault Diagnosis Process

**Figure 1:**
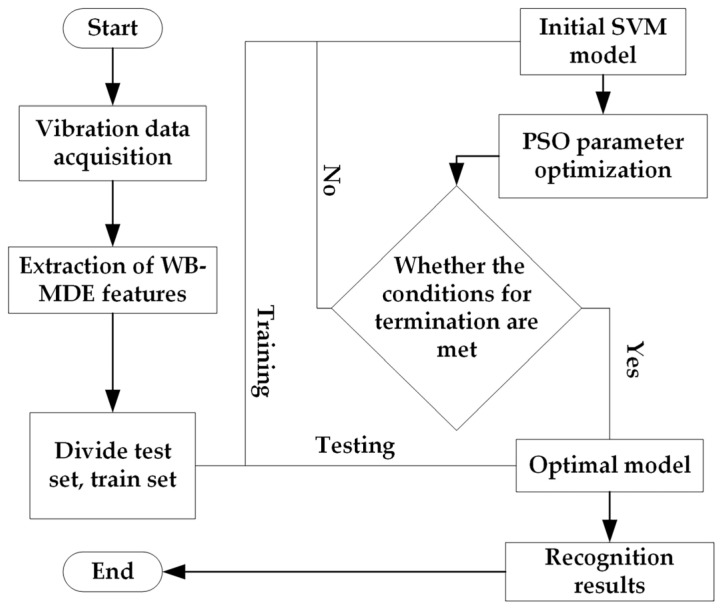
Fault diagnosis flow.

For the three-phase asynchronous motor fault signal complex problem in this paper, firstly, the vibration signal data of various fault states of the three-phase asynchronous motor are acquired via an experimental acquisition device; secondly, the feature extraction of the acquired data is carried out via WB-MDE; and, finally, the faults are classified and recognized via PSO-SVM. The diagnostic flow of this paper is shown in Figure 1.

The main process of fault diagnosis consists of the following: (1) Collecting vibration data on the three-phase asynchronous motor in four states through the experimental platform. (2) Extracting features from the vibration data through WB-MDE. (3) Dividing the dataset into a test dataset and a training dataset. (4) Using the training dataset to train the SVM model, optimizing the parameters of the SVM model via PSO, and determining whether the optimized model satisfies the termination conditions. If it meets the condition, then the output is the optimal model, otherwise, there is a return to training. (5) Using the test dataset as an input for the optimal model and examining the recognition results.

## 3. Three-Phase Asynchronous Motor Experiment

### 3.1. Experimental Data Acquisition Platform Construction

**Figure 2:**
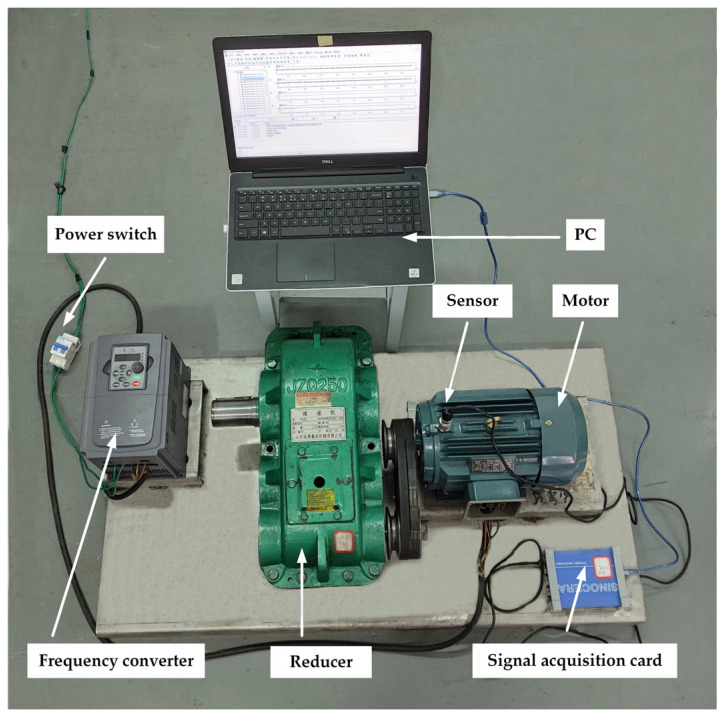
Experimental data acquisition platform.

In order to verify the effectiveness of the method proposed in this paper, a three-phase asynchronous motor experimental platform was built. The three-phase asynchronous motor fault data experimental platform consists of five parts, namely, a motor (Model: YE3-10012-4 Zhejiang Jinsu Motor Co., Ltd., Taizhou, China), frequency converter (Model: VFD9000-G5R5/P7R5-T4 Zhejiang Xintuo New Energy Co., Ltd., Jinhua, China), signal-acquisition card (Model: YE6231C JiangSu Lianneng Electronic Technology Co., Ltd., Yangzhou, China), reducer (Model: JZQ250 Shandong Zibo Xinyuan Machinery Co., Ltd., Zibo, China), and sensor (Model: CAYD051V JiangSu Lianeng Electronic Technology Co., Ltd., Yangzhou, China); the experimental data acquisition platform is shown in Figure 2.

### 3.2. Experimental Design

Fault settings: For rotor broken bars, a drill with a 4 mm bit is used to break a hole the width of three copper bars in the rotor slot of the motor. For bearing failure, a lathe tool is used to scribe damage of 1 mm in width and 0.5 mm in depth in the groove of the inner ring of the bearing at the drive end of the motor. For air gap eccentricity, the motor bearing end cover measured in connection with the rotating shaft part is ground to a thickness of 0.1 mm, half the length of the inner circle circumference. At the same time, the other half of the circle is ground into the other half of the circle pad to 0.1 mm thick, half of the length of the inner circle circumference of the copper foil. In addition, the normal state of the motor is set with a total of four operating states.

In the experiment, the frequency of the inverter regulation current is set to 40 HZ, and the corresponding motor speed is 1200 r/min. An acceleration sensor is placed above the bearing seat of the motor drive end to collect the vibration acceleration signal of the fault state [18]. The sampling frequency is 12 KHZ, the sampling time is 8 s, and the sampling interval is 2 s. For each state, 400 groups of samples are set, and each sample has 2048 points; the ratio of the test set to the training set is 3:7 [18].

### 3.3. Experimental Procedure

**Figure 3:**
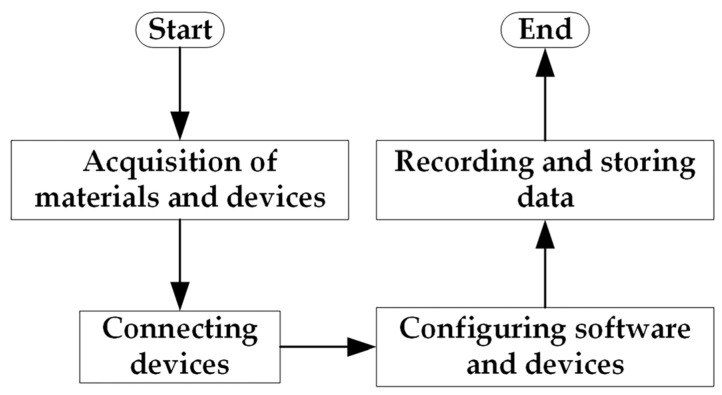
The fault diagnosis data acquisition experimental flow.

Through the three-phase asynchronous motor fault data acquisition experiment, the data related to motor faults are obtained and recorded to provide data support for fault diagnosis and prevention. The specific experimental flow of the fault diagnosis data acquisition experiment is shown in Figure 3:

(1) Experiment preparation:

Fault diagnosis equipment (sensors and data collector), target equipment (three-phase asynchronous motor and reducer), computer and data storage equipment, and experimental tools are used. The laboratory environment is safe and the work area is neat and orderly. The working status of the fault diagnosis equipment and target equipment is checked to ensure that they are functioning properly. The required software and drivers are checked to have been installed and operational.

(2) Equipment installation:

Appropriate interfaces and cables are used to connect the sensor to the target device, etc. The connection is firm, the signal is transmitted properly, and interference and errors from the surrounding environment are avoided.

(3) Data acquisition equipment and software configuration:

The data acquisition device and the corresponding software program are started. The data acquisition parameters and sampling frequency are set and the data-acquisition equipment and software are properly connected to the sensor and target device.

(4) Data acquisition and storage:

The target device is started and operated. Synchronously, the data-acquisition equipment is started and begins collecting troubleshooting data. The experiment start time and the operation status of the target device are recorded. The data acquisition software is used to display and record the collected data in real time. The data storage format and file naming rules are selected.

## 4. Analysis of Results

### 4.1. Analysis of Raw Data

**Figure 4:**
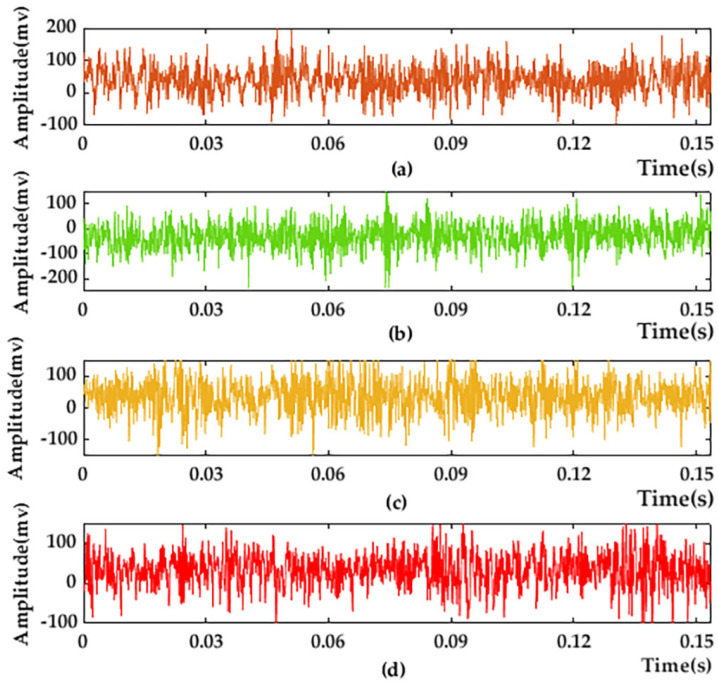
Experimental raw data: (**a**) Air gap eccentricity fault state; (**b**) normal operation state; (**c**) bearing failing fault state; and (**d**) rotor broken bar fault state.

Using the vibration data of the four motor states measured in the above experiments, one group is taken as an example; its time-domain diagram is shown in Figure 4.

Figure 4a–d corresponds to the air gap eccentricity fault state, normal operation state, bearing failing fault state, and rotor broken bar fault state of the motor, respectively. From the above figure, it can be seen that the motor vibration images under the four states are difficult to distinguish intuitively, and further data processing is required.

### 4.2. Sensitivity Analysis of WB-MDE Parameters

**Figure 5:**
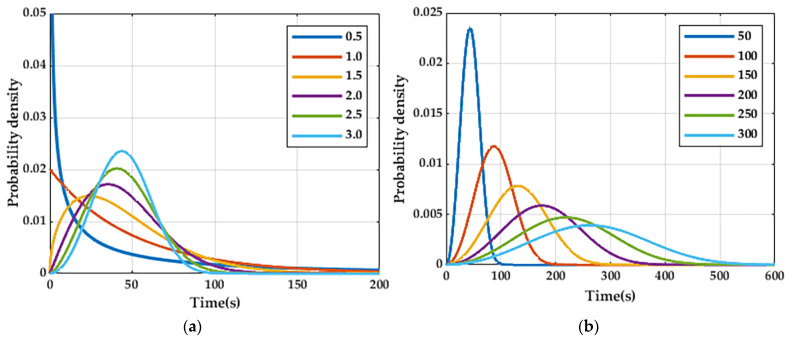
Probability density function of WB: (**a**) Probability density function of WB distribution with different shape parameters and (**b**) probability density function of WB distribution with different scaling factors.

According to the probability density function image of the Weber distribution, as shown in Figure 5, it can be seen that the shape parameter β determines the bending direction of the curvature of the image and the scaling factor η compresses the image; i.e., the larger the value, the flatter the image and the smaller the value, the steeper the image. Therefore, the scaling factor [19] η is set to 25, 50, 75, 100, and 125 and the shape parameter [20] β is set to 0.5, 1.0, 1.5, 2.0, 2.5, and 3.0 in the experiment.

**Figure 6:**
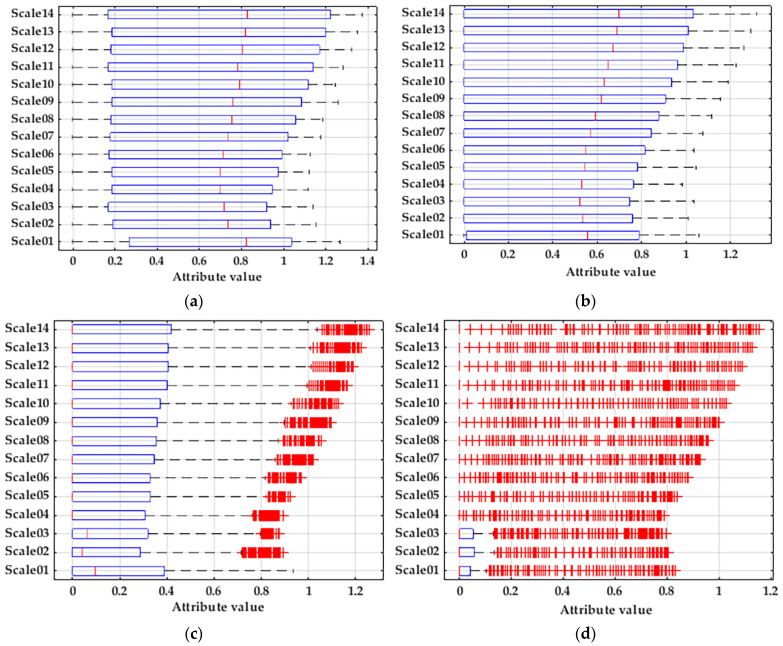

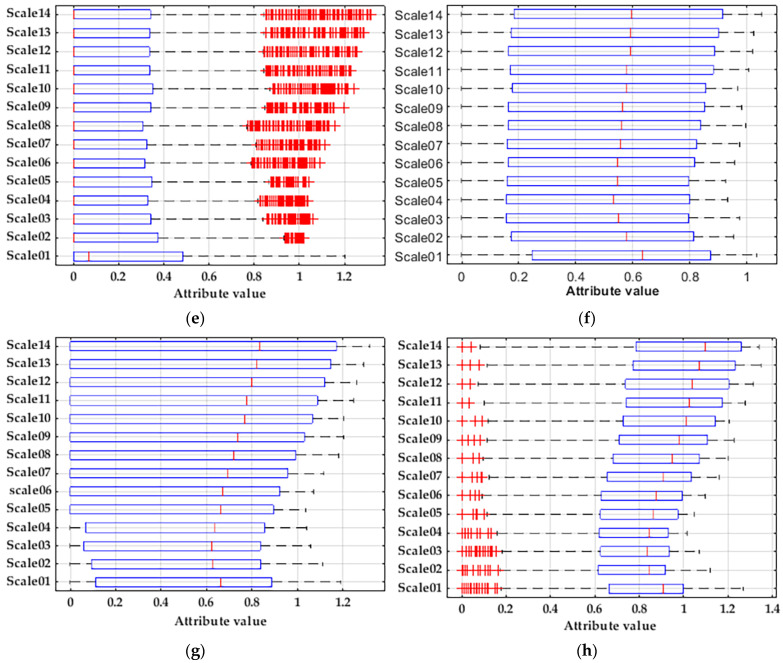
WB-MDE with different parameters: (**a**) η = 50, β = 0.5; (**b**) η = 50, β = 1; (**c**) η = 50, β = 1.5; (**d**) η = 50, β = 2; (**e**) η = 50, β = 2.5; (**f**) η = 50, β = 3; (**g**) η = 75, β = 3; and (**h**) η = 100, β = 3.

The features were extracted from the Weber distributions with different parameters. For the two groups, η = 25 and η = 125, β = 3 had no differentiation in the extraction results; the other eight groups are shown in Figure 6.

Figure 6f corresponds to the scaling factor η = 50 and the shape parameter β = 3; here, the extracted features are more effective than the other groups. By comparing the above images, the larger the scaling factor, the more dispersed the data of the same state will be and the larger the searching step for an individual will be. Additionally, a too-large searching step may transgress the optimal classification state, leading to incorrect recognition results and a low correctness rate, which affects the accuracy of the state recognition. The smaller the scaling factor, the more concentrated the data of the same state will be, which can help the classifier to reduce the classification search and accelerate the convergence of classification, but if the scaling factor is too small, it will cause the classification to fall into the local optimal state, and the correct rate is low. In order to compare the advantages and disadvantages of different parameters, the same classifier is used to recognize different groups of features, and the results are shown in Table 1.

In the table, m is the embedding dimension, t is the delay time, c is the number of categories, and scale is the scale factor. Under the same classifier, features with different parameters are extracted for the fault data of three-phase asynchronous motors. From the table, it can be seen that different parameter values will have a large impact on the final results, but it is not difficult to find out through the comparison of the data that, when the scaling factor η = 50, the training correct rate and the testing correct rate will first decrease and then increase with the increase in the shape parameter β, and when the shape parameter β = 3, the results of the correct training rate and the correct testing rate both reach 100%. When the shape parameter β = 3, the correct training rate and correct testing rate will increase and then decrease with the increase in the scaling factor η, and when the scaling factor η = 50, the results of both the correct training rate and correct testing rate reach 100%. This indicates that WB-MDE has a better classification effect for its extracted features when the scaling factor η = 50 and the shape parameter β = 3.

### 4.3. Result Analysis of Different Characteristics

**Figure 7:**
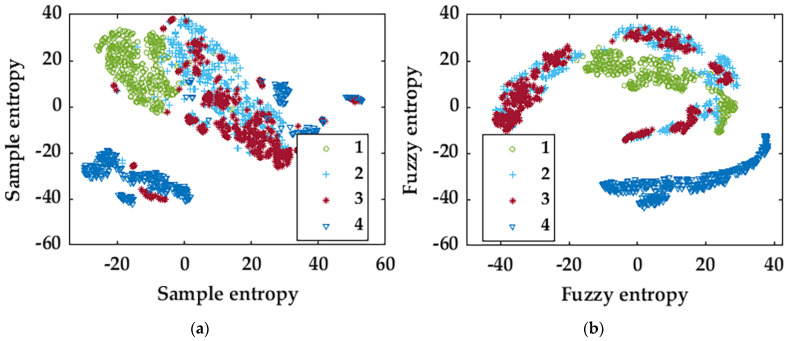

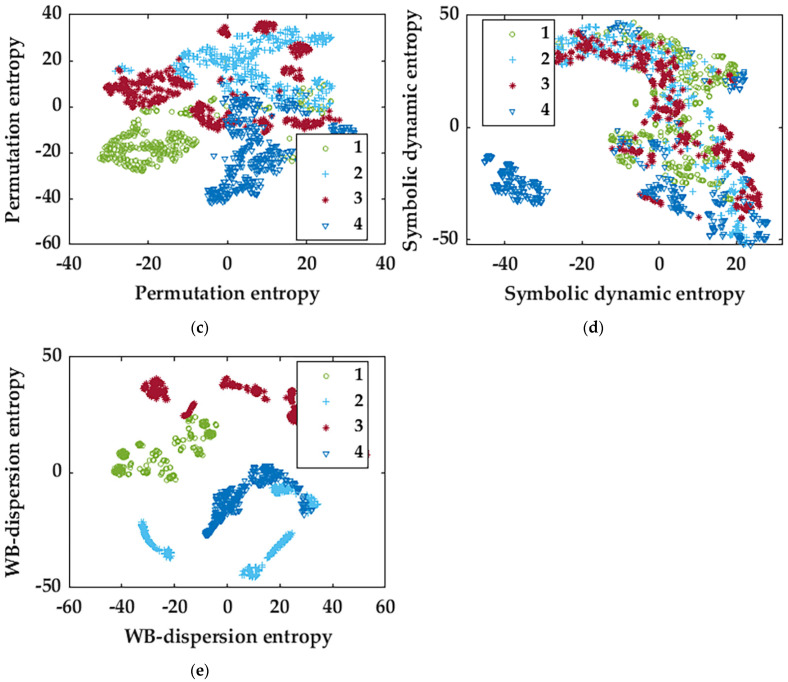
t-SNE with different characteristics: (**a**) The MSE characteristic; (**b**) the MFE characteristic; (**c**) the MPE characteristic; (**d**) the MSDE characteristic; and (**e**) the WB-MDE characteristic.

In order to verify the validity of WB-MDE feature extraction, we compare it with multiscale sample entropy (MSE) [21], multiscale fuzzy entropy (MFE) [21], multiscale permutation entropy (MPE) [22], and multiscale symbolic dynamic entropy (MSDE) [23]. Unsupervised learning is used to reduce the dimensions of feature vectors extracted in different ways. The parameters of t-SNE are set as follows [24]: the dimension of embedded space is n-components = 2, and the disorder degree is perplexity = 30. The learning rate is learning-rate = 1000, the maximum number of iterations for the optimization is n-it = 1000, and the maximum number of iterations without progress is n-it-without-progress = 30. The feature results extracted via different methods are shown in Figure 7.

Figure 7e corresponds to scaling factor η = 50, shape parameter β = 3, embedding dimension m = 2, delay time t = 1, number of categories c = 6, and scale factor scale = 14 [25], which is the best from the results of unsupervised learning, and it can be seen in the figure that only a small part of the four states of the motor is overlapping, and most of them are able to be distinguished. Compared with WB-MDE, concerning the features extracted via the other four methods, after unsupervised learning, the four fault states of the motor are not well distinguished, i.e., most of them are overlapping and only a small part of them can be distinguished.

Supervised and unsupervised learning are two common types of learning in machine learning. Since unsupervised learning does not have labeled data for training, it becomes difficult to assess the performance and quality of the model. Because there is no clear answer to compare the output of the models, it is difficult to determine whether the hidden structures or patterns learned by the models are accurate or useful. Since unsupervised learning does not have an explicit objective function to guide the learning process, the learning process of the model is relatively uncertain. This results in the model falling into local optimal solutions or failing to find meaningful structures or patterns.

In order to compare the advantages and disadvantages of different features under the supervised-learning approach, an intelligent supervised-learning approach is used to recognize different groups of features under the same PSO-SVM classifier with the parameters set as penalty coefficient-c = 1.5, kernel function coefficient-g = 1.7, number of populations ta = 20, and termination of the number of generations pop = 200 [26]; the results are shown in Table 2.

In the table, r is the similarity tolerance threshold, n is the gradient of the similarity tolerance boundary, and sigma is the standard deviation. In order to control the variables and reduce the influence of different variable values on the results, the same variable values are used for the same variables. With an embedding dimension m = 2, when the value is too large, it is easy to cause information loss; when it is too small, it is easy to cause information overlap; with scale factor scale = 14, when the value is too small, it is easy to cause insufficient feature extraction; when it is too large, it is easy to cause feature-extraction redundancy. From the result data in the table, it can be seen that the final recognition effect of WB-MDE is better than other features, and its correct training rate and correct test rate can reach 100%, in which the correct training rate is improved by 2.71–16.88% compared to other methods, and the correct test rate is improved by 4.74–13.93% compared to other methods. Multiscale sample entropy is biased, highly mutable, and lacks continuity. There is too little differentiation in multiscale fuzzy entropy and complexity and invalidity in the calculation of multiscale permutation entropy. Furthermore, multiscale symbolic dynamic entropy is too small to be of use.

### 4.4. Analysis of Results for Different Classifiers

Using the WB-MDE feature, we compare the K nearest neighbor classifier, random forest classifier, BP neural network classifier, SVM classifier, and PSO-SVM classifier [27], and obtain the recognition accuracy under different classifiers, as shown in Table 3.

The performance of the classifiers is compared under different classifiers using the same features extracted via WB-MDE. From the correct recognition rates in the table, it can be seen that all the results can reach more than 96% when the features extracted via WB-MDE are used to recognize the fault states of the motor. For the K nearest neighbor classifier, its shortcomings are that the selection of Numneighbors is prone to overfitting or underfitting; the random forest classifier is not able to give predictions beyond the training dataset and the output cannot be continuous; the BP neural network classifiers are prone to overfitting and low accuracy; and the support vector machine classifier requires the manual input of the parameters and has low efficiency. By comparing with the above classifiers, the PSO-SVM classifier can perform parameter optimization to avoid manual selection and its recognition correctness can reach 100%, which is improved by 1.05–3.34% compared to other classifiers.

**Figure 8:**
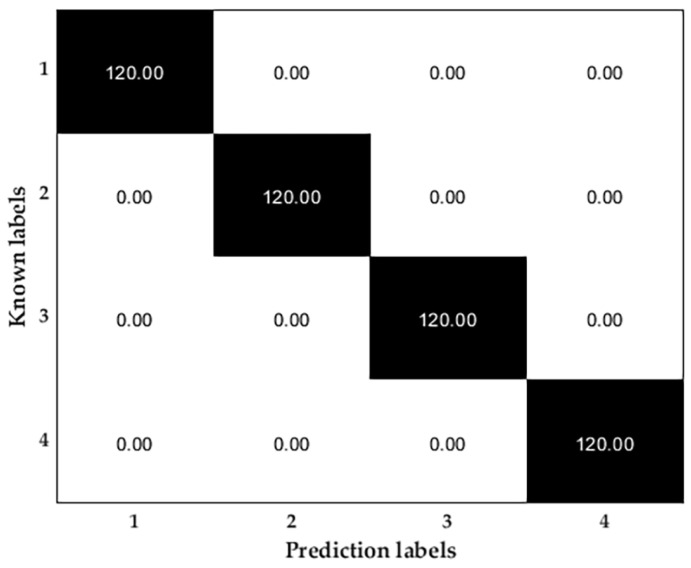
Confusion matrix for test data.

Based on the three-phase asynchronous motor fault diagnosis method combining multiscale Weibull dispersion entropy and a particle swarm optimization support vector machine, for each state, 400 groups of samples are set, and each sample has 2048 points, finally, the ratio of the test set to the training set is 3:7. The identification of the four motor states is shown in Figure 8.

## 5. Noise Resistance and Generalization Test

### 5.1. Noise-Resistance Test

**Figure 9:**
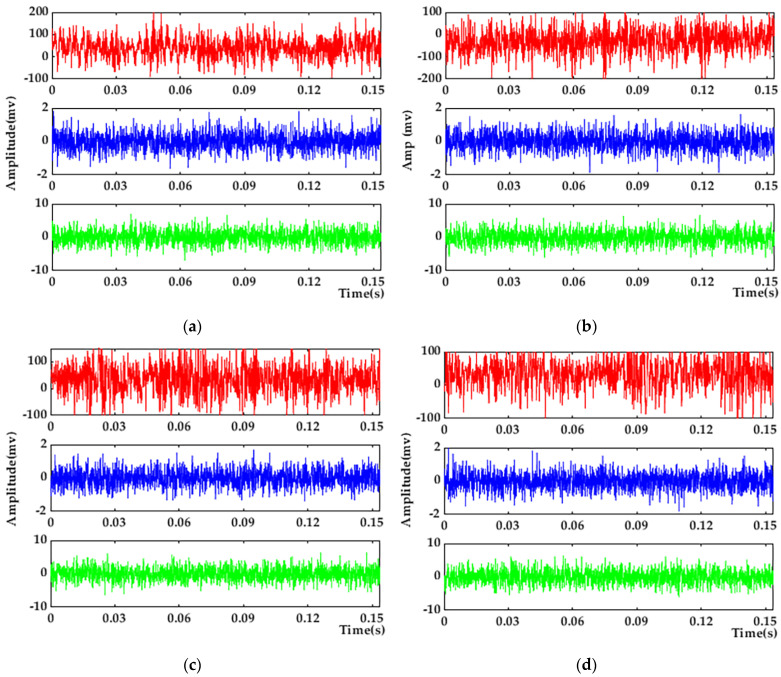
The differences of different signal-to-noise ratios: (**a**) air gap eccentricity fault state; (**b**) normal operation state; (**c**) bearing failing fault state; and (**d**) rotor broken bar fault state.

In the actual working environment of the three-phase asynchronous motor, there is noise due to the harsh conditions, complex environment, and the existence of connections between different devices. In order to be close to the actual motor working environment and to test the noise resistance of the method proposed in this paper, Gaussian white noise with a different signal-to-noise ratio (SNR) is added to the original vibration data of the motor. The formula is as follows:

(21) $$SNR=10\mathrm{lg}\frac{p_{s}}{p_{n}}$$

where $p_{s}$ is the energy of the original signal and $p_{n}$ is the energy of the noise signal. Gaussian white noise with signal-to-noise ratios of 6 dB and −6 dB is added to the original data. Using the four motor states’ original data and the difference data after adding Gaussian white noise, one of the groups is taken as an example and is shown in Figure 9.

**Figure 10:**
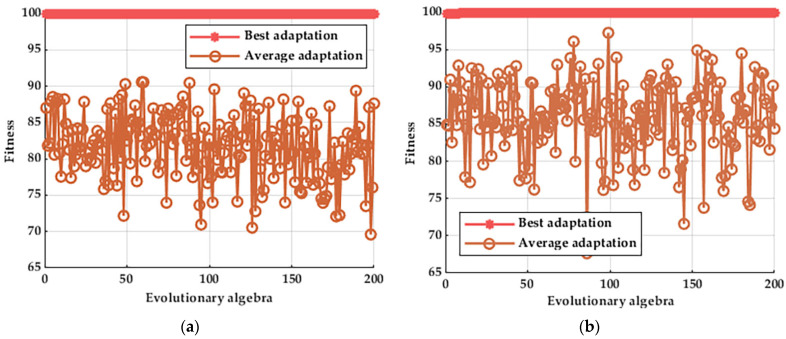
Adaptability of different signal-to-noise ratios: (**a**) a signal-to-noise ratio of −6; (**b**) a signal-to-noise ratio of 6.

From the above figure, it can be observed that the larger the signal-to-noise ratio, the smaller the difference compared to the original data (after adding noise), and, in contrast, the smaller the signal-to-noise ratio, the larger the difference compared to the original data (after adding noise). However, when observing the image, it can be seen that the trend of the image after adding Gaussian white noise remains similar to the original data, so the authenticity of the data can be maintained. The data are then brought into the model to recognize the fault state of the motor, and the adaptation curve is shown in Figure 10.

From the above figure, it can be seen that at an SNR of −6 dB, the value of the average adaptation is slightly lower than the case of an SNR of 6 dB, which is due to the high ratio of the Gaussian white noise signal to the original signal [28], but in terms of the final correctness of the test, 100% can still be achieved with the method proposed in this paper.

### 5.2. Generalizability Test

In order to validate the generalizability of the method proposed in this paper [29], motor-vibration data publicly available under the CHIST-ERA SOON project in CHIST-ERA III—European Coordinated Study on Long-Term ICT and ICT-Based Scientific Challenges (768977) are used. Three types of motor-state data are measured via the Y-direction sensor and X-direction sensor of the extracted dataset, 100 sets of samples are set for each state, and each sample contains 2048 data points [30]; the ratio of the test set to the training set is 3:7. The first motor state is normal operation, no load, and a motor rotational speed half of the maximal rotational speed; the second state is the motor with mechanical failure of the rotational shaft imbalance, no load, and a motor rotational speed half of the maximal speed; the third state is the motor with an electrical fault, the fault resistance is 50 Ω, no load, and the motor speed is half of the maximum speed [31].

**Figure 11:**
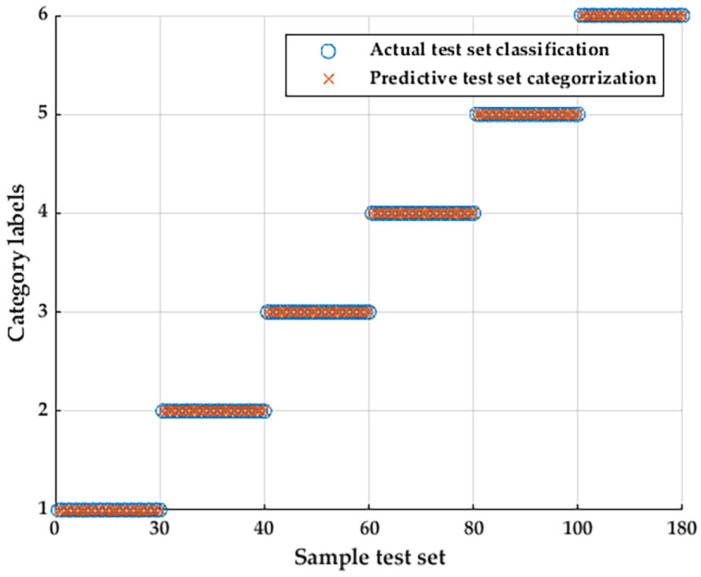
The test result of the open data.

Using the method proposed in this paper, the above data are used to classify and recognize the motor faults, and the recognition results are shown in Figure 11.

As can be seen from the graph of the results, the recognition result of the open motor data is also 100%, which indicates that the method proposed in this paper has high generalization and meets the test requirements.

## 6. Conclusions

Aiming at the problems of insufficient feature extraction, unstable recognition effect, insufficient generalization ability, and noise resistance in the health monitoring and intelligent fault diagnosis of three-phase asynchronous motors, a three-phase asynchronous motor fault diagnosis method based on the combination of multi-scale Weber dispersion entropy and a particle swarm optimization support vector machine is proposed and the following conclusions are drawn through experimental analysis:

(1) The multi-scale Weber dispersion entropy proposed in this paper can fully and comprehensively mine the fault information of the three-phase asynchronous motor fault state, overcome the problem of nonlinear and non-smooth fault signals, obtain more sensitive information about the operating state of a motor, quantify the regularity of the data features, and improve the utilization rate of the original data and the stability of the classifiers.
(2) The proposed model in this paper has good noise immunity and generalization for three-phase asynchronous motor fault diagnosis. By adding Gaussian white noise with different signal-to-noise ratios to the original data and utilizing public datasets for detection, the model indicates broad and steady applications.

In the future, the health monitoring and intelligent fault diagnosis of three-phase asynchronous motors will be intensively researched so as to obtain a fault diagnosis model with better noise resistance and generalization ability.

## Figures and Tables

**Table 1:** Correct rate for different parameters.

Serial Number	η	β	Classifier	Train Accuracy Rate	Test Accuracy Rate
a	50	0.5	Pso-svm m = 2 t = 1 c = 6 scale = 14	95.08%	94.58%
b	50	1.0		89.79%	89.79%
c	50	1.5		81.25%	81.25%
d	50	2.0		61.04%	61.04%
e	50	2.5		70.20%	70.20%
f	50	3.0		100%	100%
g	25	3.0		0	0
h	75	3.0		97.65%	97.29%
i	100	3.0		96.33%	96.45%
j	125	3.0		0	0

**Table 2:** Correct rate for different characteristics.

Features	Classifier	Parameters	Training Correctness	Test Correctness
MSE	PSO-SVM	m = 2 scale = 14 r = 0.15	93.54%	92.85%
MFE		m = 2 scale = 14 r = 5 n = 2	97.29%	95.26%
MPE		m = 2 scale = 14 t = 1	96.45%	94.55%
MSDE		m = 2 scale = 14 sigma = 12	83.12%	86.07%
WB-MDE		m = 2 scale = 14 t = 1 c = 6 η = 50 β = 3	100%	100%

**Table 3:** Correct rate for different classifiers.

Classifiers	Parameters	Recognition Correctness
K Nearest Neighbor Classifier	Numneighbors = 10	97.70%
Random Forest Classifier	Ntree = 1	97.91%
BP Neural Network Classifier	Ir = 0.1 mnist = 0.1	97.80%
SVM Classifier	−c = 1.5 −g = 1.7	96.66%
PSO-SVM	−c = 1.5 −g = 1.7 pop = 20 ta = 200	100%

## Data Availability

The data used to support the findings of this study are available from the corresponding authors upon request.
